# Comparison of Clinical Outcomes between Newly Diagnosed and Pre-Existing Diabetes Mellitus Patients after Acute Coronary Syndrome

**DOI:** 10.2174/0115701611322555250219111038

**Published:** 2025-04-27

**Authors:** Wei-Chieh Lee, Huang-Chung Chen, Chih-Yuan Fang, Yi-Hsuan Tsai, Yun-Yu Hsieh, Tien-Yu Chen, Yen-Nan Fang, Po-Jui Wu, Hsiu-Yu Fang, Ping-Yen Liu

**Affiliations:** 1 School of Medicine, College of Medicine, National Sun Yat-sen University;; 2 Division of Cardiology, Department of Internal Medicine, Chi Mei Medical Center, Tainan, Taiwan;; 3 Institute of Clinical Medicine, College of Medicine, National Cheng Kung University, Tainan, Taiwan;; 4 Division of Cardiology, Department of Internal Medicine, Kaohsiung Chang Gung Memorial Hospital, Chang Gung University College of Medicine, Kaohsiung, Taiwan;; 5 Biostatistics Center, Kaohsiung Chang Gung Memorial Hospital, Kaohsiung, Taiwan;; 6 Division of Cardiology, Department of Internal Medicine, Jen-Ai Hospital, Taichung, Taiwan;; 7 Division of Cardiology, Department of Internal Medicine, National Cheng Kung University Hospital, College of Medicine, National Cheng Kung University, Tainan, Taiwan

**Keywords:** Acute coronary syndrome, diabetes mellitus, newly diagnosed, pre-existing, HbA1c

## Abstract

**Aims:**

This study aimed to evaluate clinical outcomes, including recurrent acute coronary syndrome (ACS) and mortality, in ACS patients with varying HbA1c levels, addressing the controversy over optimal targets in those with newly diagnosed and pre-existing diabetes mellitus (DM).

**Methods:**

From January 2005 to December 2019, a total of 33,990 patients were identified with ACS in the Chang Gung Research Database based on their medical history. After excluding patients without DM and baseline or subsequent HbA1C data, a cohort of 11,870 DM patients was divided into two groups: one consisting of 6,089 patients with newly diagnosed DM and the other comprising 5,781 patients with pre-existing DM.

**Results:**

During the three-year follow-up, the pre-existing DM group experienced worse clinical outcomes, such as increased rates of re-ACS, major bleeding, cardiovascular (CV) events, and all-cause mortality. Optimal HbA1c levels for mitigating re-ACS and/or CV mortality and all-cause mortality appeared to differ between the two DM cohorts. Re-ACS and CV mortality reached their highest at an HbA1c of 6.8% for all DM patients, 6.6% for newly diagnosed, and 6.7% for pre-existing cases. The greatest all-cause mortality risk was at an HbA1c of 7.4% for all DM patients, 7.0% in newly diagnosed, and 8.2% in pre-existing patients.

**Conclusion:**

Upon comparing newly diagnosed DM patients with those with pre-existing DM, a poorer prognosis was observed in the latter group, attributed to older age and a higher burden of comorbidities. Throughout the follow-up period, maintaining consistently low HbA1c levels did not reduce the incidence of re-ACS nor enhance survival rates.

## 1 BACKGROUND

Type 2 diabetes mellitus (DM) is widely recognized as a significant economic and healthcare burden in real-world practice, with its prevalence gradually increasing worldwide [1-5]. DM plays a crucial role in the development of atherosclerosis, a pathological condition that is a major contributor to cardiovascular (CV) diseases [6-9]. The progression of this pathophysiological process is largely influenced by long-term blood glucose levels [8, 10, 11]. A higher HbA1c level has been identified as a potential indicator of in-hospital mortality in patients with acute coronary syndrome (ACS) [12, 13]. In individuals with DM, each 1% increase in HbA1c is associated with an estimated 13% rise in the risk of CV events [14, 15]. The necessity for stricter blood glucose control in DM patients remains a topic of debate. Various clinical guidelines suggest aiming for an HbA1c level below 7.0% or 6.5% in DM management [8, 16, 17]. Conversely, another guideline suggests that a less stringent control target (HbA1c level below 8.0%) may also be beneficial for patients experiencing microvascular or macrovascular complications [18]. The comparison of clinical outcomes between patients with newly diagnosed DM and those with pre-existing DM after ACS, as well as the optimal target for HbA1c, remains insufficiently explored. In this context, we conducted a comprehensive cohort study to evaluate the risk of recurrent ACS (re-ACS), CV events, and all-cause mortality among patients with newly diagnosed and pre-existing DM after an ACS event over a three-year follow-up period.

## 2 METHODS

### 2.1 Patient Population

Between January 2005 and December 2019, a total of 33,990 patients with a diagnosis of ACS and DM were enrolled. Their medical histories were extracted from the research database of Chang Gung Memorial Hospital, Taiwan's largest healthcare network. We published the research on ACS using the research database of Chang Gung Memorial Hospital [19-22].

Individuals aged 18 years and older who were diagnosed with ACS, as classified by the International Classification of Diseases, Ninth Revision, Clinical Modification (ICD-9-CM) codes 410.xx, 411.xx, and 412.xx or Tenth Revision (ICD-10) codes I20, I21, and I22, in conjunction with DM, coded as ICD-9-CM 250.xx or ICD-10 E10, E11, were eligible for inclusion in this study. Excluding those without DM and lacking baseline or subsequent HbA1c data, a total of 11,870 patients were selected for the analysis. This group was then divided into two subgroups: 6,089 patients with DM newly diagnosed at the time of ACS and 5,781 with pre-existing DM. We collated and compared data regarding general demographics, comorbidities, medication usage, re-ACS, CV mortality, and all-cause mortality between the two DM groups.

### 2.2 Ethical Statement

This retrospective study received ethical approval from the Institutional Review Committee at Kaohsiung Chang Gung Memorial Hospital, with the approval number 202101103B0. This endorsement confirms adherence to the ethical guidelines set forth in the 1975 Declaration of Helsinki.

### 2.3 Definitions

Newly diagnosed DM was defined as a fasting plasma glucose level ≥ 126 mg/dL on two separate occasions and an HbA1c level ≥ 6.5% during hospitalization for this ACS [23, 24]. Blood glucose levels, including HbA1c and fasting plasma glucose, were measured within 48 hours of the ACS episode. Pre-existing DM referred to patients with a prior history of DM who were already under medical management. Re-ACS was defined as any subsequent episode of acute myocardial ischemia, including conditions like unstable angina, non-ST-segment elevation myocardial infarction, or ST-segment elevation myocardial infarction. Major bleeding was defined as Bleeding Academic Research Consortium 3 or 5 bleeding after an ACS episode [25-27]. CV mortality was classified as any death resulting from CV causes, and all-cause mortality included deaths due to any reason.

### 2.4 Study Endpoints

The study's endpoints were focused on examining the occurrence of re-ACS, major bleeding, mortality due to CV reasons, and mortality arising from any cause.

### 2.5 Statistical Analyses

Data is reported as mean values with standard deviations or as frequencies (percentages). Clinical characteristics were compared between the two groups using independent samples t-tests for continuous variables and chi-square or Fisher's exact tests for categorical variables. The influence of HbA1c levels on the incidence of re-ACS, CV mortality, and all-cause mortality in patients with newly diagnosed and pre-existing DM was assessed using Cox proportional hazards models. These models were adjusted for age and comorbid conditions to calculate hazard ratios. To compare the rates of re-ACS, CV mortality, and all-cause mortality over a one-year follow-up period between the groups, Kaplan-Meier survival analysis with the log-rank test was carried out. A p-value below 0.05 was deemed to indicate statistical significance. SAS software version 9.4 (SAS Institute Inc., Cary, NC, USA) was used for all statistical calculations.

## 3 RESULTS

### 3.1 Comparison of Baseline Characteristics between Patients with Newly Diagnosed and Pre-existing DM

Between the groups with newly diagnosed DM and those with pre-existing DM (Table **1**), the newly diagnosed DM patients were younger (66 ± 12.38 years *vs*. 71 ± 11.58 years; p<0.001), predominantly male (68.02% *vs*. 57.48%; p<0.001), and had a higher body mass index (BMI) (25.59 ± 4.24 kg/m^2^ *vs*. 24.87 ± 4.55 kg/m^2^; p<0.001). The newly diagnosed DM group also had fewer comorbidities, except for a higher prevalence of smoking (15.82% *vs*. 5.79%; p<0.001). This group exhibited a higher baseline HbA1c level (8.14 ± 2.00% *vs*. 7.54 ± 1.74%; p<0.001) and a greater prevalence of HbA1c levels above 7.5% (44.88% *vs*. 35.34%; p<0.001). Additionally, the use of antiplatelet agents, including aspirin, clopidogrel, and ticagrelor, was more common in the newly diagnosed DM group. This group also had a longer follow-up period.

### 3.2 Kaplan-Meier Curve Analysis of Re-ACS, Major Bleeding, CV Mortality, and All-cause Mortality

**Fig. (1):**
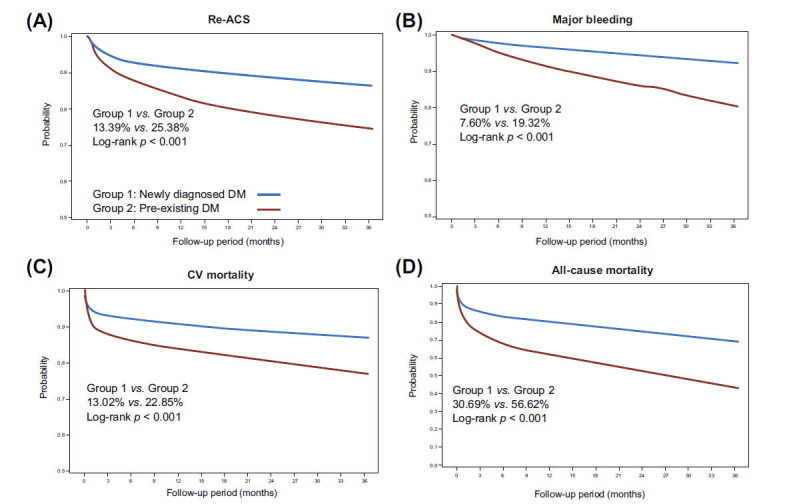
Kaplan-Meier curve analysis of re-ACS, major bleeding, CV mortality, and all-cause mortality between those with newly diagnosed DM and pre-existing DM. (**A**). Kaplan-Meier curve analysis of re-ACS was significantly higher in the pre-existing DM group than the newly diagnosed DM group during the three-year follow-up period (13.39% *vs*. 25.38%; log-rank p<0.001). (**B**). Kaplan-Meier curve analysis of major bleeding was significantly higher in the pre-existing DM group than the newly diagnosed DM group during the three-year follow-up period (7.60% *vs*. 19.32%; log-rank p<0.001). (**C**). Kaplan-Meier curve analysis of CV mortality was significantly higher in the pre-existing DM group than the newly diagnosed DM group during the three-year follow-up period (13.02% *vs*. 22.85%; log-rank p<0.001). (**D**). Kaplan-Meier curve analysis of all-cause mortality was significantly higher in the pre-existing DM group than the newly diagnosed DM group during the three-year follow-up period (30.69% *vs*. 56.62%; log-rank p<0.001). **Abbreviations:** ACS: acute coronary syndrome; CV: cardiovascular; DM: diabetes mellitus.

Between those with newly diagnosed DM and pre-existing DM (Fig. **1**), the Kaplan-Meier curve analysis of re-ACS (13.39% *vs*. 25.38%; log-rank p<0.001), major bleeding (7.60% *vs*. 19.32%; log-rank p<0.001), CV mortality (13.02% *vs*. 22.85%; log-rank p<0.001), and all-cause mortality (30.69% *vs*. 56.62%; log-rank p<0.001) was significantly higher in the pre-existing DM group than the newly diagnosed DM group at three-year follow-up period.

### 3.3 The Comparison of Clinical Outcomes between Following HbA1C <7.5% and ≥7.5% in Newly Diagnosed and Pre-existing DM Population

In the comparison of strict versus less stringent glucose control among newly diagnosed DM patients, no significant differences were observed in clinical outcomes, including re-ACS, major bleeding events, CV mortality, and all-cause mortality at both one-year and three-year follow-up periods (Table **2**). Conversely, in the population with pre-existing DM, a higher incidence of major bleeding was observed with strict glucose control compared to less stringent glucose control at the one-year follow-up (8.3% *vs*. 6.4%; p=0.046). Additionally, a higher incidence of all-cause mortality was noted with strict control at the three-year follow-up (38.1% *vs*. 32.5%; p=0.001).

### 3.4 The Comparison of Clinical Outcomes between Changes in HbA1C in Newly Diagnosed and Pre-existing DM Population

In the population of newly diagnosed DM, a significantly higher incidence of major bleeding was observed in patients with both baseline and subsequent HbA1c levels below 7.5% during a one-year follow-up period (Table **3**). Additionally, a significantly higher incidence of major bleeding was observed in patients whose baseline HbA1c was below 7.5% but rose to 7.5% or higher during a three-year follow-up period.

In the population with pre-existing DM, there was only a trend toward a lower incidence of all-cause mortality observed in patients who maintained both baseline and subsequent HbA1c levels above 7.5% during a three-year follow-up period.

### 3.5 Cox Proportional Hazards Models of Subsequent HbA1C Level on the Rates of re-ACS and/or CV Mortality and All-cause Mortality Among Patients with Newly Diagnosed DM and those with Pre-existing DM

A Cox proportional hazards regression model was employed to assess the association between subsequent HbA1c levels and clinical outcomes, such as re-ACS and/or CV mortality, as well as all-cause mortality, as depicted in Fig. (**2**). Optimal outcomes for re-ACS and/or CV mortality were observed at an HbA1c level of 6.8% in the overall population, 6.6% in the newly diagnosed DM group, and 6.7% in the pre-existing DM group. Optimal outcomes for all-cause mortality corresponded to an HbA1c level of 7.4% in the overall diabetic population, 7.0% in the group with newly diagnosed DM, and 8.2% in the group with pre-existing DM.

## 4 DISCUSSION

In this study, individuals with newly diagnosed DM demonstrated improved clinical outcomes, including reduced occurrences of re-ACS, major bleeding, CV mortality, and all-cause mortality, in comparison to those with pre-existing DM. These differences might be explained by the older age and greater prevalence of comorbidities among the pre-existing DM group. In terms of glycemic control, no significant differences in clinical outcomes were observed between patients with newly diagnosed DM who had less stringent glycemic control (subsequent HbA1c ≥7.5%) and those with stricter control (subsequent HbA1c <7.5%). However, in the pre-existing DM group with less stringent glycemic control, a significantly lower incidence of all-cause mortality was noted after three years. Analyzing the impact of changes from baseline to subsequent HbA1c levels revealed that stricter glycemic control did not necessarily lead to better overall outcomes. Nevertheless, more stringent control of subsequent HbA1c may be associated with the most favorable results in terms of re-ACS and/or CV mortality, but it did not yield the best outcomes for all-cause mortality. The optimally subsequent HbA1c goal for reducing the risk of re-ACS and/or CV mortality in patients with concurrent ACS and DM was 6.8%. The optimal HbA1c goal for reducing the risk of all-cause mortality in patients with concurrent ACS and DM was 7.4%.

### 4.1 The Optimal HbA1C Level for re-ACS in DM Populations

Patients with DM who have undergone ACS require extra focus due to their increased risk of in-hospital major adverse cardiac events and elevated long-term mortality rates [28-32]. Our research indicated that those with pre-existing DM faced a higher rate of re-ACS, major bleeding events, CV mortality, and mortality from all causes compared to those with newly diagnosed DM. While achieving optimal glucose levels has been shown to reduce microvascular complications, its effectiveness in mitigating macrovascular complications and decreasing long-term mortality appears limited in this particularly vulnerable group [33-36]. In our study, aiming for a strict HbA1c target appeared to yield better outcomes in terms of re-ACS and/or CV mortality. However, this approach did not extend the same benefit to all-cause mortality in both newly diagnosed and pre-existing DM patients. Previous studies have suggested that minor incremental improvements in microvascular outcomes can be achieved with A1C values approaching normal, prompting providers to consider recommending lower A1C goals than the standard <7% for patients with a short duration of diabetes, long life expectancy, and no significant CV disease, while advising less stringent targets for those at risk of severe hypoglycemia, with limited life expectancy, advanced vascular complications, significant comorbidities, or difficulties in meeting standard goals despite comprehensive management [37, 38]. In the European Society of Cardiology ACS guidelines, there is no specified treatment goal for patients with DM following an ACS [39]. According to the recommendations of recent *American Diabetes Association* guidelines, the treatment goal for HbA1c is set at 7.0% [40]. Based on clinical practice experience and the recommendations from various guidelines, we adopted a cutoff point for HbA1c at 7.5% [41, 42]. In our study, an optimal HbA1c level of less than 7% was associated with a lower risk of re-ACS and/or CV mortality in both newly diagnosed and pre-existing DM groups. However, for the outcome of all-cause mortality, the optimal HbA1c level was less in the pre-existing DM group. Strict glycemic control might be less beneficial in this group due to factors, such as older age and a higher prevalence of comorbidities.

### 4.2 Strict Sugar Control and Mortality

A meta-analysis revealed that meticulous blood glucose management decreases the likelihood of certain macrovascular and microvascular complications, yet it does not influence overall mortality rates [43]. Furthermore, maintaining stricter control over glucose levels can lead to an increased incidence of severe hypoglycemia, a condition where blood sugar levels drop dangerously low [44, 45]. Additional research highlights that particularly strict blood sugar management aimed at preventing CV disease may increase mortality risk, especially among older individuals [46-50]. These findings suggest a nuanced approach to setting glycemic targets; for individuals newly diagnosed with diabetes, a more stringent HbA1C goal may be beneficial, whereas, for those who have been living with the condition, a more relaxed target could be advisable to minimize risks and improve overall outcomes. In recent years, the introduction of innovative medications, such as Sodium-Glucose Cotransporter-2 inhibitors (SGLT2i) and Glucagon-Like Peptide-1 Receptor Agonists (GLP1RA), has marked a significant advancement in the treatment of DM and CV disease [51-65]. These drugs have been particularly noted for their positive effects on CV outcomes in patients with ACS [66-68]. Both classes of medications not only aid in glycemic control but also offer protective benefits against CV events, making them a valuable addition to the therapeutic arsenal against diabetes and its complications [69-71]. Therefore, it is important to consider individualizing the optimal HbA1C target and the selection of medications for DM management, tailoring the approach to meet each patient's unique needs and circumstances [72-75].

## 5 STUDY LIMITATIONS

This study faced certain constraints typical of a retrospective design, as it relied solely on data extracted from medical records. The classification of patients was greatly dependent on the use of ICD-9M and ICD-10M codes, which are subject to interpreting physician's clinical judgment. The codes and recording biases limit the ability to draw causal inferences. Additionally, the study did not investigate the influence of varying severities of coronary artery disease, nor did it examine left ventricular function, heart failure incidents, or the effects of emerging DM treatments, such as SGLT2 inhibitors and GLP1 receptor agonists. We were unable to provide detailed clinical outcomes, including heart failure events and the CV impact of new DM treatments. Due to the observational nature of the study, we could not investigate the underlying mechanisms explaining why different HbA1c levels affected clinical outcomes, particularly the observed differences between newly diagnosed and pre-existing DM groups. We also excluded patients who lacked baseline or subsequent HbA1c levels, as this may influence the outcomes of this cohort study. The study population consisted primarily of East Asian individuals, which may limit the generalizability of the findings to other ethnic groups with different genetic backgrounds and lifestyle factors. Despite these limitations, the study offers valuable insights into the optimal HbA1C targets and provides a comparison of more stringent versus more relaxed DM management strategies among East Asian individuals with diabetes following ACS. Different HbA1c goals and DM treatment strategies may be necessary for patients with newly diagnosed and pre-existing DM following ACS. Further research, particularly large-scale randomized controlled trials, is needed to validate these findings and to comprehensively explore the optimal HbA1c targets for different patient subgroups, including those with newly diagnosed and pre-existing DM. After ACS, the DM treatment strategy should not only focus on achieving target HbA1c levels but also consider the additional benefits of new DM treatments.

## CONCLUSION

Upon comparing newly diagnosed DM patients to those with pre-existing DM, a poorer prognosis was observed in the latter group, attributed to older age and a higher burden of comorbidities. Throughout the three-year follow-up period, maintaining consistently low HbA1c levels did not reduce the incidence of re-ACS nor enhance survival rates. Further validation of these findings necessitates a large-scale randomized study.

## AUTHORS’ CONTRIBUTIONS

The authors confirm their contribution to the paper as follows: study conception and design: P-JW; data collection: H-CC, C-YF, T-YC, Y-NF, H-YF; data analysis and interpretation: Y-HT, Y-YH; writing the paper: W-CL and final editing: P-YL. All authors reviewed the results and approved the final version of the manuscript.

## Figures and Tables

**Fig. (2):**
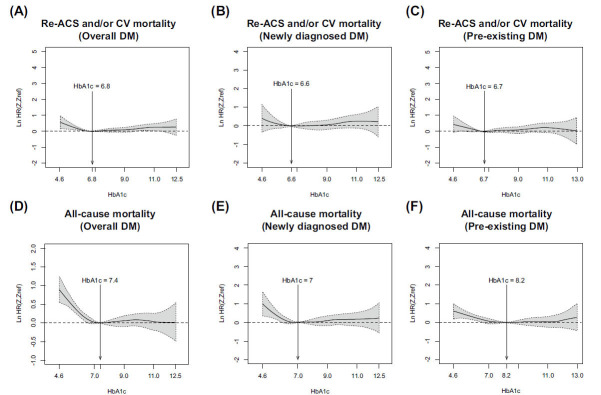
Cox proportional hazards models of following HbA1C level on the rates of re-ACS and/or CV mortality and all-cause mortality among patients with newly diagnosed DM and those with pre-existing DM. **A-C**. Optimal outcomes for re-ACS and/or CV mortality were observed at an HbA1c level of 6.8% in the overall population (**A**), 6.6% in the newly diagnosed DM group (**B**), and 6.7% in the pre-existing DM group (**C**). **D-F**. Optimal outcomes for all-cause mortality corresponded to an HbA1c level of 7.4% in the overall diabetic population (**D**), 7.0% in the group with newly diagnosed DM (**E**), and 8.2% in the group with pre-existing DM (**F**). **Abbreviations:** ACS: acute coronary syndrome; CV: cardiovascular; DM: diabetes mellitus; HbA1c: hemoglobin A1c.

**Table 1:** Baseline characteristics in the patients with newly diagnosed and pre-existing DM.

-	Newly Diagnosed	Pre-existing	P value
***Number***	6089	5781	-
***General demographics***	-	-	-
Age (years)	66 ± 12.38	71 ± 11.58	<0.001
Male sex (%)	4142 (68.02)	3323 (57.48)	<0.001
BMI (kg/m^2^)	25.59 ± 4.24	24.87 ± 4.55	<0.001
***Comorbidities***	-	-	-
Hypertension (%)	3932 (64.58)	3918 (67.77)	<0.001
PAOD (%)	44 (0.72)	84 (1.45)	<0.001
COPD (%)	217 (3.56)	318 (5.50)	<0.001
ESRD (%)	328 (5.39)	1016 (17.57)	<0.001
Smoking (%)	963 (15.82)	335 (5.79)	<0.001
Liver cirrhosis (%)	49 (0.80)	183 (3.17)	<0.001
Prior GI bleeding (%)	413 (6.78)	1191 (20.60)	<0.001
Prior stroke (%)	247 (4.06)	834 (14.43)	<0.001
***HbA1C (%)***	-	-	-
Baseline (%)	8.14 ± 2.00	7.54 ± 1.74	<0.001
Baseline ≥ 7.5% (%)	2733 (44.88)	2043 (35.34)	<0.001
***Medication***	-	-	-
Antiplatelet agent	-	-	-
Aspirin (%)	4355 (71.52)	3274 (56.63)	<0.001
Clopidogrel (%)	3551 (58.32)	2938 (50.82)	<0.001
Ticagrelor (%)	963 (15.82)	539 (9.32)	<0.001
***F/U period (years)***	2.2 ± 1.1	1.6 ± 1.2	<0.001

**Note: ** Data are expressed as mean (standard deviation) or as number (percentage).

**Abbreviations:** DM: diabetes mellitus; BMI: body mass index; PAOD: peripheral arterial occlusive disease; COPD: chronic obstructive pulmonary disease; ESRD: end stage renal disease; GI: gastrointestinal; *F/U: follow-up.*

**Table 2:** The comparison of clinical outcomes between following HbA1C <7.5% and ≥7.5% in newly diagnosed and pre-existing DM population.

-	Newly Diagnosed		-	Pre-existing		*-*
Following HbA1C	<7.5%	≥7.5%	P value	<7.5%	≥7.5%	P value
One-year	-	-	-	-	-	-
Re-ACS	9.0%	9.6%	0.529	17.4%	18.5%	0.426
Major bleeding	3.4%	2.7%	0.222	8.3%	6.4%	**0.046**
CV mortality	1.7%	2.1%	0.368	4.3%	4.0%	0.678
All-cause mortality	4.9%	5.4%	0.490	13.4%	11.6%	0.134
Three-year	-	-	-	-	-	-
Re-ACS	13.3%	13.6%	0.789	26.2%	27.7%	0.348
Major bleeding	7.9%	7.1%	0.359	19.8%	17.7%	0.138
CV mortality	4.9%	5.0%	0.889	12.0%	10.0%	0.079
All-cause mortality	14.9%	14.2%	0.547	38.1%	32.5%	**0.001**

**Note: ** Data are expressed as percentage.

**Abbreviations:** DM: diabetes mellitus; HbA1C: hemoglobin A1c; ACS: acute coronary syndrome; CV: cardiovascular.

**Table 3:** The comparison of clinical outcomes between different change of HbA1C in newly diagnosed and pre-existing DM population.

**-**	**Newly diagnosed**				**-**	**Pre-existing**				***-***
The change of HbA1C	<7.5% ➜ <7.5%	<7.5% ➜ ≥7.5%	≥7.5% ➜ <7.5%	≥7.5% ➜ ≥7.5%	P value	<7.5% ➜ <7.5%	<7.5% ➜ ≥7.5%	≥7.5% ➜ <7.5%	≥7.5% ➜ ≥7.5%	P value
One-year	-	-	-	-	-	-	-	-	-	-
Re-ACS	9.4%	10.1%	8.7%	9.0%	0.847	17.7%	20.3%	15.9%	17.6%	0.420
Major bleeding	4.0%	2.1%	2.2%	2.5%	**0.038**	8.1%	7.6%	7.4%	5.9%	0.581
CV mortality	1.8%	2.2%	1.5%	2.3%	0.587	4.5%	4.6%	3.8%	3.7%	0.828
All-cause mortality	5.0%	6.4%	4.8%	5.3%	0.730	13.4%	14.6%	12.2%	10.4%	0.352
Three-year	-	-	-	-	-	-	-	-	-	-
Re-ACS	13.6%	14.1%	12.8%	13.1%	0.905	26.6%	28.8%	24.8%	27.2%	0.578
Major bleeding	8.4%	10.1%	6.4%	5.9%	**0.019**	20.3%	21.4%	17.0%	16.3%	0.115
CV mortality	5.2%	6.5%	4.2%	4.9%	0.386	12.7%	10.6%	10.5%	9.8%	0.306
All-cause mortality	15.3%	18.5%	13.5%	13.0%	0.078	37.8%	37.4%	37.6%	30.3%	0.060

**Note: ** Data are expressed as percentage.

**Abbreviations:** DM: diabetes mellitus; HbA1C: hemoglobin A1c; ACS: acute coronary syndrome; CV: cardiovascular.

## Data Availability

Data from this study can be obtained from the corresponding authors upon request. Partially, this research utilized data from the Chang Gung Research Database, courtesy of Chang Gung Memorial Hospital. The analyses, interpretations, and conclusions drawn in this study do not reflect the views of Chang Gung Memorial Hospital.

## LIST OF ABBREVIATIONS

ACS: Acute coronary syndrome
DM: Diabetes mellitus
CV: Cardiovascular
re-ACS: Recurrent ACS
ICD-9-CM: International classification of diseases, ninth revision, clinical modification
SGLT2i: Sodium-glucose cotransporter-2 inhibitors
GLP1RA: Glucagon-like peptide-1 receptor agonists

## STANDARDS OF REPORTING

STROBE guidelines were followed.
